# Arhuaco indigenous women’s memories and the Colombian Truth Commission: methodological gaps and political tensions

**DOI:** 10.1007/s42597-021-00062-4

**Published:** 2021-08-31

**Authors:** Juliana González Villamizar, Ángela Santamaría, Dunen Kaneybia Muelas Izquierdo, Laura María Restrepo Acevedo, Paula Cáceres Dueñas

**Affiliations:** 1 Justus-Liebig-University Gießen, Licher Straße 76, 35394 Gießen, Germany; 2 Instituto CAPAZ, Bogota, Colombia; 3 Centro de Paz, Conflictos y Posconflictos, Intercultural School for Indigenous Diplomacy, Universidad del Rosario, Carrera 24, 63C-69 Bogotá, Colombia; 4 Technical Secretary to the National Commission of Indigenous Women, Intercultural School for Indigenous Diplomacy (EIDI), Universidad del Rosario, Carrera 24, 63C-69 Bogotá, Colombia; 5 Intercultural School for Indigenous Diplomacy (EIDI), Universidad del Rosario, Carrera 24, 63C-69 Bogotá, Colombia

**Keywords:** Truth commissions, Indigenous peoples, Women and gender, Intersectionality, Intercultural methodologies, Wahrheitskommissionen, Indigene Völker, Frauen und Gender, Intersektionalität, Interkulturelle Methoden

## Abstract

The Truth, Peaceful Coexistence, and Non-Repetition Commission (CEV) is one of the transitional justice mechanisms contained in the peace agreement signed between the Colombian government and the Revolutionary Armed Forces of Colombia (FARC) guerrilla in 2016. The CEV mainstreams gender and ethnic differential approaches and is also the first to actively deploy intersectionality as a framework to approach violence committed against women of ethnic groups. The article draws on a decolonial and intercultural perspective to analyze the challenges that the CEV faces to make visible Indigenous women’s experiences and agencies during the armed conflict. Based on participatory research conducted with Arhuaco women of the Sierra Nevada de Santa Marta to produce a report to the CEV, the article shows the methodological gaps that exist between Arhuaco women’s approaches to memory and the Truth Commission’s methodological framework. The article also argues that the Commission’s strategy to confront political dynamics within Indigenous communities that marginalize women’s processes further deepens these gaps and contributes to invisibilize their voices in this scenario.

## Abstract (de)

Die Kommission für Wahrheitsfindung, friedliches Zusammenleben und Gewährleistung der Nichtwiederholung (Comisión para el Esclarecimiento de la Verdad, la Convivencia y la No Repetición [CEV]) ist einer der Transitional-Justice-Mechanismen aus dem Friedensabkommen zwischen kolumbianischer Regierung und FARC-Guerrilla von 2016. Die CEV wendet differenzielle Ansätze bezüglich Gender und ethnischer Abstammung an und nutzt zudem erstmals aktiv das Konzept der Intersektionalität als Rahmen, um Gewalt gegen Frauen aus ethnischen Gruppen zu untersuchen und anzugehen. Der Beitrag bedient sich einer dekolonialen und interkulturellen Perspektive, um die Herausforderungen zu analysieren, vor denen die CEV bei dem Unterfangen steht, Erfahrungen und Wirken indigener Frauen während des bewaffneten Konflikts sichtbar zu machen. Basierend auf einer partizipativen Studie mit Arhuaco-Frauen aus der Sierra Nevada de Santa Marta, durchgeführt für einen Bericht an die CEV, zeigt der vorliegende Artikel die methodische Kluft zwischen den Ansätzen von Arhuaco-Frauen in Bezug auf das Erinnern und dem methodischen Rahmenwerk der Wahrheitskommission. Der Beitrag legt auch dar, dass die Strategie der Kommission, um politische Dynamiken innerhalb indigener Gemeinschaften, die die Prozesse von Frauen marginalisieren, anzugehen, die Kluft weiter vertieft und dazu beiträgt, die Stimmen der Frauen unter diesen Umständen ungehört zu machen.

## Introduction

The peace agreement signed between the Colombian government and the Revolutionary Armed Forces of Colombia (FARC) in 2016 established the Truth, Coexistence, and Non-Repetition Commission (CEV) as one of the bastions of the transitional justice architecture to be implemented in this country. The mechanism is designed to bring Colombian society the truth about the violent events that occurred in over fifty years of armed conflict. As an extra-judicial investigation commission, the CEV is also a “vehicle of historical memory” (Jaramillo 2014). By the end of its three-year mandate, the CEV must present a final report of its findings along with its recommendations to the Colombian government. According to its regulatory decree, the CEV must guarantee the broad and pluralistic participation of individual and collective victims (Presidencia de la República 2017).

Beginning in the early 1980s, truth commissions have become one of the mechanisms most often employed to deal with the consequences of armed conflict and political violence. Transitional justice was initially associated with judicial and institutional reform through legal and criminal prosecutions. The political transitions in Latin America and especially the establishment of the National Commission on the Disappearance of Persons (CONADEP) in Argentina produced a shift away from jurisprudence towards democratization and civil renewal based on the concept of restorative justice (Oettler 2006; Fobear 2014). Thus, rather than prosecutions, truth commissions focus on victims’ needs for recognition, rehabilitation and reparation (Minow 1998; De Gamboa Tapias 2020). According to Kimberly Theidon, the explicit goal of truth commissions is “the writing of new national narratives that are more inclusive of groups that are historically marginalized within the nation-state” (Theidon 2006, p. 457). It is by these means that truth commissions expect to prevent further violence and human rights abuses in the future (Olsen et al. 2010).

Given their focus on victims, truth commissions aim to guarantee that all social sectors and especially minority groups participate effectively from the beginning of the process and are active on all levels of decision making (Sing’Oei and Young 2011). In the 1990s and 2000s, several events propelled gender mainstreaming in transitional justice and an increased participation of women in peace processes. These included the declaration made by the International Criminal Tribunal for the Former Yugoslavia (ICTY) that rape and sexual slavery are crimes against humanity; the 1995 UN Conference on Women in Beijing, which pressed conflict resolution instruments to cast light on women’s experiences and to break the silence on sexual violence; and the UN Security Council Resolution 1325. These transformations were intended to remedy common gender biases and shortcomings of transitional accountability mechanisms. Fionnualla Ní Aólain and Catherine Turner point, for instance, to truth commissions’ usual definition of violations, which tend to exclude home and familial contexts and lack acknowledgement of the historical and structural roots of gender-based violence in relation to the patriarchal domination of pre-existing armed conflicts (Ní Aoláin and Turner 2007). Analyzing the role of gender and women’s issues in the truth commissions of South Africa, Guatemala, Peru, Sierra Leone and Liberia, Jeremy Sarkin and Sarah Ackermann stress the importance of hiring enough carefully selected women commissioners and staff, and for recommendations to address women’s economic, social and cultural structures and realities as a means to guarantee their access and meaningful exertion of civil and political rights in the post-conflict period (Sarkin and Ackermann 2019).

Moreover, the work of truth commissions in Guatemala and Canada highlighted the importance of addressing ethnic and racial discrimination and including the concerns of Indigenous groups in the clarification process. The Guatemalan Historical Clarification Commission (CEH) made a demonstrable contribution to the participation of Indigenous people in public life by declaring that the state had committed acts of ethnocide against the Mayan population (Oettler 2006). The Canadian Truth and Reconciliation Commission (TRC) acted as an alternative tribunal to channel the conflict between First Nations and the Canadian state as it focused on the physical and psychological abuse, cultural appropriation, forced assimilation, and the destruction of First Nations’ languages, identities and cultures that occurred at the Indian Residential Schools beginning in 1880 (Flisfeder 2010; Nagy 2014).

Thanks to feminist and lesbian, gay, bisexual, transexuell/transgender and intersexual (LGBTI) activism during the peace negotiations, the CEV must pay special attention to the victimization of women by mainstreaming a gender approach in all its activities (Presidencia de la República 2017). Moreover, as demanded by Indigenous and Afro-descendant groups at prior consultation with transitional institutions in 2019, the CEV incorporates an Ethnic Peoples Directorate (EPD) in charge of the Commission’s approach to ethnic communities. The CEV is also the first to include an intersectional perspective in its methodology to deal with violence committed against women of ethnic groups. However, similarly to previous truth commissions, in its early stages, the CEV has had to tackle significant difficulties in addressing the priorities and concerns of Indigenous women. According to Pascha Bueno-Hansen, the Peruvian Truth and Reconciliation Commission (PTRC) also struggled to include factors such as language, ethnicity and culture as relevant to the analysis of gender as the “entrenched legacies of colonialism manifest in the division of knowledge production in Lima and data gathering in the branch offices” (Bueno-Hansen 2015, p. 69). Emily Rosser contends that the CEH failed to work out the interrelationship between gender and ethnicity, as it denied that systematic wartime rape is constitutive of genocide (Rosser 2007). Although the TRC declared domestic violence against Indigenous women to be a product of colonial, sexist and racist relationships, it did not recognize the need to address these harms, nor to recommend structural reforms to family laws and services to transform such violence.

There are various obstacles to Indigenous women’s participation in the construction of narratives about the armed conflict, which thus make it difficult for them to influence the work and outcomes of truth-seeking processes. Many of them wrap their grief, shame and fears in silent dignity or try to heal them through rituality and ancestral practices beyond the framework of Occidental modernity. Indigenous women often also struggle to play a role in state scenarios that privilege the experience and knowledge of Indigenous male leaders. Recent feminist participatory-action research in Guatemala and Colombia shows that supporting Indigenous women’s memory processes in ways that honour their cosmological reference points and account for the historical deprivations and subordination they have endured requires the implementation of complex and expensive strategies in their territories. Lengthy and flexible time frames are also necessary to build trust and construct methodologies that respect Indigenous women’s means of dealing with past violence. Ángela Santamaría shows how sustained processes of caring exchange and artistic practices with Uitoto women help construct collective memories of the violence of the rubber regime in the Colombian Amazon that centre these women’s agency, resistance and solidarity practices (Santamaría 2017). For two decades, Alison Crosby and Brinton M. Lykes also used creative methodologies with Maya Ixil, K’iche’, Kaqchikel, Mam and Chuj women who survived sexual violence during the armed conflict in Guatemala as resources for psycho-emotional healing and to support these women’s struggles for truth, justice and reparation (Crosby and Lykes 2019).

This article analyzes the challenges that the CEV faces in making visible Indigenous women’s experiences and agencies during the armed conflict based on the perspective of Arhuaco women of the Sierra Nevada de Santa Marta in the Colombian Caribbean region. Despite continuous attacks to their cultural and physical existence, the Arhuaco people hold on strongly to their ancestral cosmological and spiritual principles. Arhuaco women in particular lead an unprecedented resistance process to revitalize their traditional knowledge and to enact their political and spiritual role in the community. As an intercultural team, we prepared the “*ZAKU SEYNEKUN ZUN NOKWUZANAMU*: Voices of Mother Earth” report together with these women to support their participation in the CEV. Based on this experience, the article reveals the gaps that Arhuaco women perceive between the Truth Commission’s methodological framework and the cosmological and epistemological reference points of their approach to memory and working through traumatic events. Arhuaco women assert that speaking of past violence and digging deeper into painful experiences is alien to the traditional practices of the Arhuaco people and does not contribute to strengthening Arhuaco women’s process of cultural and epistemic resistance. The article also demonstrates that the Commission’s strategy to deal with political dynamics within Indigenous communities that marginalize women’s processes further deepens these gaps and contributes to obscuring their voices in this context.

After explaining the methods employed in the research leading to this article, we provide a background on the continuum of colonial and armed conflict violence historically inflicted on the Arhuaco people, and the CEV’s research task. We then present the contemporary process of Arhuaco women and their expectations regarding the report and their participation at the CEV. Based on the extensive collaborative work of the Intercultural School for Indigenous Diplomacy (EIDI) with Arhuaco women and two collectively constructed information gathering exercises, the remainder of the article examines the methodological gaps and the political tensions Arhuaco women perceive in terms of the Truth Commission’s clarification work. While recognizing the efforts of CEV staff to enable Indigenous women’s participation in the truth-seeking process, the article presents an initial productive critique of the CEV contribution in this regard based on the collective reflections of Arhuaco women and people.

## Research methods

This article is the result of permanent dialogue between ongoing research into the incorporation of intersectionality at the CEV and the work of the EIDI team—which includes an Arhuaco woman who is also co-author of this article—with Arhuaco women over several years. The team uses popular education (Freire 2002) and participatory action-research (Ortiz and Borjas 2008) methods to exchange and produce knowledge collaboratively with Indigenous women. These methods are based on local practices and everyday scenarios and ensure equal participation opportunities for everyone (Mejía 2014; Borjas et al. 2015). The approach also fosters reflective processes to identify and understand the impact of the team’s interventions in Indigenous communities affected by violence and armed conflict (Anderson and Olson 2003; Bush 2009; Lopera-Molano and Lopera-Molano 2020). These activities are part of the intercultural diploma courses, which offer informal education intended to strengthen Indigenous women’s political processes within their communities. The methodologies are co-created between the team and Indigenous women and include making music, dancing, performing traditional rituals and using academic research tools. One of the diploma courses held in October 2019 focused on strengthening the Arhuaco women’s organization process and was attended by fifty Arhuaco women and fifty Arhuaco men.

### The “*ZAKU SEYNEKUN ZUN NOKWUZANAMU”*

Voices of Mother Earth’ report mentioned above is based on the activities of the 2019 intercultural course and materials gathered over ten years of collaborative work with the Arhuaco people. The report presents the CEV with an account of violence against Arhuaco women that highlights its continuity since colonial times and their resistance practices across time. The intercultural methods used as part of this research have enabled the team to centre Arhuaco women’s knowledge and practices while simultaneously constructing the memory of long-term violence against them, beginning with the institutionalized racism of the colonial practices of the Capuchin Mission and its boarding school in San Sebastian de Rabago, and intensifying with the inferiorizing practices used by armed actors during the armed conflict. The report analyzes sexual violence, forced displacement, forced recruitment of women and children, forced assimilation, labour exploitation, loss of food autonomy and the impact of legal and illegal extractive practices in the territory of the Sierra Nevada de Santa Marta. It also demonstrates that although this continuum of violence deeply affects traditional female knowledge, Arhuaco women’s efforts to preserve and revitalize it are fundamental for its survival. The final version of the report was agreed upon with Arhuaco women and traditional authorities in Umuriwa in March 2020 and was presented publicly to the CEV in July 2020. Due to the restrictions in place because of the Covid-19 pandemic, the event took place in one of the Commission’s virtual meeting rooms and was streamed online.^1^

Research on the incorporation of intersectionality at the CEV involves interviews with truth commissioners and staff members, participatory observation of public events and meetings with civil society since the beginning of the Commission’s mandate and the review of methodological and analytical documents produced by various working groups within the institution. As a researcher at Instituto CAPAZ, which provides consultancy to the CEV, one of the co-authors of this article facilitated and accompanied the presentation of the report. Two meetings were held with lead CEV researchers to adjust the report to the Commission’s guidelines for submission and to prepare its public presentation. The data analysis is based on the examination of the content of personal communications, interviews and primary documents.

## The continuum of colonial and armed conflict violence against the Arhuaco people and the CEV’s research task

The Sierra Nevada de Santa Marta is an isolated system of the Andes in north-eastern Colombia inhabited by the Arhuaco, Kogi, Kankuamo and Wiwa Indigenous groups. According to Bastian Bosa, during the 20th century, the education of Arhuaco people was marked by logics of assimilation and the forced separation of Arhuaco children from their parents to exterminate their culture and family relationships (Bosa 2020). In 1917, Capuchin missionaries opened a boarding school in San Sebastian de Rabago, the capital municipality for Arhuaco people, to “civilize and evangelize” them. Bosa (2020) argues that the missionary education for the reduction and assimilation of Indigenous people by the Church and by delegation of the Colombian state “performed as a centrepiece of the nation’s project for the disintegration of Indigenous families and communities” (Bosa 2020, p. 1). The San Sebastian de Rabago boarding school was closed in 1982, as the Arhuaco people expelled the Capuchin Mission through non-violent protest.

Ángela Santamaría shows that internment at the boarding school was intended to discipline and dominate girls’ and young women’s bodies through physical violence, punishments, sexual abuse and the prohibition of the traditional Arhuaco knowledge and practices (Santamaría 2021). This violence left its mark on the Arhuaco people for several generations, affecting women, families and children alike. Resentment, painful memories and feelings of racial inferiority are daily reminders that during that period, Arhuaco women and their traditional knowledge were considered “primitive”. In response to decades of cultural and spiritual assimilation, Arhuaco women decided to revitalize their spiritual and political role in the community in a process led by Alcira Izquierdo as Woman, Family and Childhood Committee coordinator of the Tayrona Indigenous Confederation (CIT), the Arhuaco people’s main representative body.

**Fig. 1:**
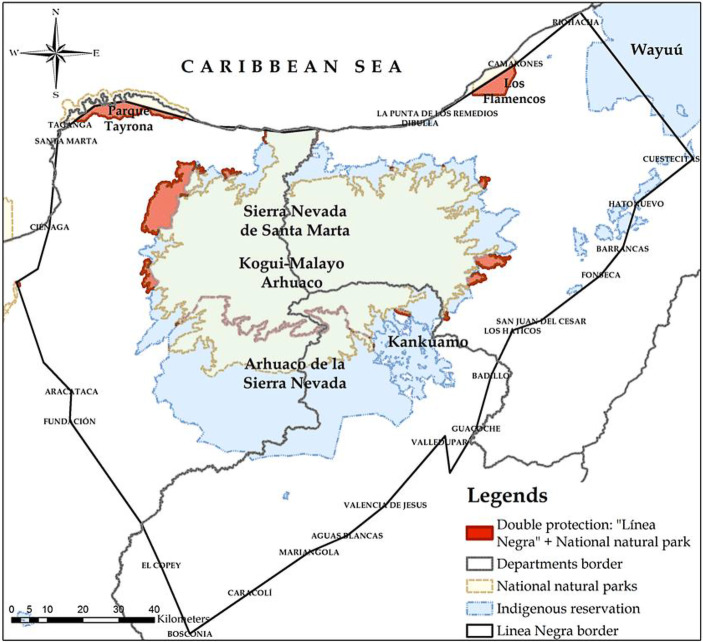
Map of the Sierra Nevada de Santa Marta in north-eastern Colombia. (Pérez et al. 2017)

Violent practices of forced assimilation against Arhuaco women continued through the actions of different armed actors in the Sierra Nevada since 1980. The FARC established the 19th Front in the Sierra Nevada in the 80s. The National Liberation Army (ELN) established their presence in the region in 1987. In 1988, the United Self-Defence Forces of Colombia (AUC) entered the Sierra Nevada to combat the escalation of kidnappings and extortion by the ELN against the nascent mining industry (Ronderos 2016). The group implemented terror strategies including massacres, forced displacement and kidnappings of guerrilla family members, leaving more than 72,000 victims in the department of Cesar (CNMH 2016, p. 13). Local politicians, cattle ranchers, farmers, businessmen and traders directly or indirectly supported the AUC (ibid.). Guerrilla groups and the AUC used the territories within the “Black Line”—a political delimitation agreed on between the Arhuaco people and the Colombian government in 1973 that contains the sacred sites of the Indigenous people of the Sierra Nevada—to control the entrance to the region, traffic illegal weapons, build escape routes and hide the kidnapped (Fig. 1). In 2003, under President Álvaro Uribe’s policy of democratic security, the military’s *Popa* and High Mountain Battalions were established in the Sierra to recover territories under the FARC’s control. Confrontations among armed actors in the region affected Arhuaco women profoundly, especially due to forced displacement and the forced recruitment of Indigenous youth.^2^

The peace agreement signed between the Colombian government and the FARC in 2016 decrees the reincorporation of combatants and the implementation of the Comprehensive System of Truth, Justice, Reparation and Non-Repetition (SIVJRNR), of which the CEV is part. In 2016, the National Indigenous Organization of Colombia (ONIC) and the National Afro-Colombian Peace Council (CONPA), two important ethnic organizations, established the Ethnic Commission for Peace and the Defence of Territorial Rights to join the negotiation table in Havana and were able to ensure the inclusion of an Ethnic Chapter encompassing a gender, woman, family and generation safeguard. The latter was incorporated as a provision that draws on the collective knowledge of Indigenous women, who proposed the woman, family and generation approach to influence public policy for differentiated attention for women, children and older persons. At the Ethnic Commission, both Indigenous and Afro-descendant women used the gender, woman, family and generation safeguard to assert their shared cosmological reference points and guarantee their participation in the peace process and the implementation of differentiated measures for women belonging to ethnic groups. Other women’s groups and feminist and LGBTI organizations joined forces to press for gender mainstreaming in all the agreements, which was achieved via the creation and placement at the negotiation table of a gender subcommission made up of female representatives of the Colombian government and FARC. These efforts successfully led to the CEV incorporating a gender unit from the very beginning of its mandate: the Gender Working Group (GWG).

According to Presidential Decree 588 of 2017, which contains the CEV’s mandate, the institution’s main objective is “to satisfy the right to truth, which the Final Agreement signed between the Colombian government and the FARC acknowledges as the cornerstone for the consolidation of peace” (art. 1). The CEV must clarify and foster recognition of thirteen aspects of the armed conflict, including crimes against human rights and international humanitarian law and their human and social impact on several social groups, including women, children and adolescents (id., art. 11). This work has been distributed among nine research nuclei dealing with the relationship between democracy and armed conflict; state responsibilities; armed actors and other actors responsible for violations; economic dynamics, land dispossession and forced displacement; illegal drugs, economy and armed conflict; coping and non-violent resistance strategies and peaceful transformations; causes, dynamics and impact of the armed conflict on ethnic peoples; international dimensions and exile; and society, culture and armed conflict. The CEV must also clarify the historical context, the multiple origins and causes of the armed conflict and the factors and conditions that facilitate or contribute to its persistence.

The CEV clarifies patterns of violence based on data about the conflict gathered from individual and collective testimonies, cases and reports submitted by organizations and collectives, participative diagnoses and public dialogues, and it constructs illustrative cases of each of the identified patterns. These patterns are supplemented with explanatory contexts from historical, political, economic, cultural and environmental perspectives (Comisión para el Esclarecimiento de la Verdad and la Convivencia y la No Repetición 2019). The CEV also implements a territorial approach that focuses on the actors and dynamics in different parts of the country, the population in exile and four differential approaches: gender, ethnic, psychosocial as well as course of life and disability. The CEV established working groups to be in charge of each differential approach. While the GWG supports the clarification and recognition of crimes committed against women and LGBTI people by mainstreaming a gender approach, the EPD oversees the ethnic approach, which focuses on racial discrimination against Indigenous, Afro-descendant, Raizal, Palenquero and Rom people. This Directorate was requested by Indigenous and Afro-descendant organizations during the prior consultation with ethnic peoples in 2019 to guarantee that the Commission addresses ethnic issues approached from a level of higher autonomy, and ensures a permanent and horizontal dialogue with ethnic peoples throughout its mandate. Patricia Tobón Yagarí, a young Embera lawyer elected as truth commissioner at the CEV, leads the Commission’s work with ethnic groups.

The EPD and ethnic peoples developed an “ethnic methodology” stating that the documentation process and methodological design of the CEV must be agreed upon with ethnic traditional authorities and women belonging to ethnic groups, “in order to consider their experiences and knowledge, as well as those of their communities and organizations, with regard to the documentation of cases and information-gathering in their contexts” (Comisión para el Esclarecimiento de la Verdad and la Convivencia y la No Repetición 2018b, p. 9). It also considers oral sources as fundamental to recognizing cultural diversity and “value forms of production, narration, and dissemination of [ethnic groups’] knowledge and experience by means of the cultural and artistic expressions manifest in their worldviews” (id., 12). The ethnic methodology also incorporates intersectionality as an approach related to the gender, woman, family and generation approach. Initially, the GWG was in charge of clarifying the truth about violence against Indigenous women. However, during the earliest months of the CEV’s term of office, disagreements arose between the GWG and Indigenous women regarding the meaning of family from the perspective of gender analysis and of the political struggle behind the woman, family and generation approach (González Villamizar and Bueno-Hansen 2021). As a result, at the SIVJRNR’s prior consultation with Indigenous peoples, the National Commission of Indigenous Women (CNMI), an instance of the Government’s Permanent Roundtable with Indigenous Peoples and Organizations (MPC), pressed for representation within the EPD. In September 2019, almost one year after the beginning of its mandate, the CEV hired a representative of the Indigenous Women’s Commission to oversee the incorporation of the gender, woman, family and generation approach for Indigenous women.

## Arhuaco women’s “sowing” of recognition as holders of political and spiritual power

During the intercultural diploma courses, Arhuaco women insisted that the body of the Arhuaco woman is a human-scale representation of the territory of the Sierra Nevada de Santa Marta (Santamaría 2021). Intellectual and political leader Ati Quigua affirms that Mother Earth (*Ati Seynekun*) is the principle, foundation and sustaining life force, as well as the identity and essence of the *Iku*^3^ (Quigua 2017). The Arhuaco woman is the symbolic representation of Mother Earth; she is the bearer of life and considered the mother of humanity (Quigua 2017) responsible for the multiple moral and sacred facets of spiritual harmony and the maintenance of balance for the Arhuaco people and the universe. As expressed by Seydin Rosado, leader of Nabusímake:

According to the Law of Origin (*Sein Zare*), women support the spiritual work of the *Mamos*^4^. The wives of a *Mamo*, for example, eat special food and live quietly in their homes, preparing the materials for the spiritual work. They also engage in prolonged fasting. In the Law of Origin, at dawn woman was made of bright gold, and to remain gold, she had to refrain from sexuality. After having sex with her husband, the woman is no longer made of gold and becomes of marble (personal communication, 2016).

The Law of Origin represents the birth of Indigenous peoples in their territory and provides each Indigenous group with an understanding of their cosmology and norms of conduct according to natural law. Despite restrictions in the Arhuaco Law of Origin in terms of sexuality, diet and work to support the *Mamos*, participants in the courses reflect on the role of women in Arhuaco self-government and spiritual practices. A tension usually emerges between participants affirming that only some Arhuaco women have the spiritual permission to govern, and others—very close to the process Alcira Izquierdo leads—who state that women governed in the past, and therefore Mother Earth has to be consulted in order to update this right.

Contemporary Arhuaco women engage in spiritual work to “sow” their recognition as holders of spiritual and political power and the role they may play in self-government. Pastor and Santamaría explain that this form of self-recognition before the territory constitutes a specific methodology of political-spiritual litigation and consultation through which participants will emerge as political-spiritual agents who have resisted multiple forms of violence and are allowed to govern according to the Law of Origin (Pastor and Santamaría 2021). In this respect, more than four Women’s Community Assemblies and women’s *pagamentos*^5^ have been held in different parts of the Sierra Nevada (Quigua 2017). They employ other traditional methodologies such as spiritual fasting, the *marneykuan*^6^, sacred dances and visits to feminine sacred sites, as well as communal reflections on the history of Arhuaco women and their role in Arhuaco self-government. These activities also serve to revitalize feminine knowledge and transmit it to younger generations.

Each Assembly is attended by approximately 500 people and takes place under the guidance of one of the Major *Mamos* of the Sierra Nevada: *Mamo* Kingumwu Niño of the Major Kankurwa^7^ of Seykwinkuta in Nabusímake. He guides the process and serves as the spiritual interpreter in consultation with Mother Earth at the *Kadukwu*^8^ and in female sacred sites such as *Ati Serecha* (Quigua 2017). Alcira Izquierdo highlights the importance of *Mamo* Kingumwu’s support of the women’s process. As a spiritual leader, he grew up not eating salt and due to his discipline and compliance with the Law of Origin, he has been able to dance the *tani* or Dance of Truth. For the Arhuaco people, processes have to be supported both materially and in terms of *tikurigun *or spiritually. *Mamo* Kingumwu is the best “lawyer” or interpreter of the Law of Origin so that the women’s process can be “sown” spiritually and materially. *Mamo *Kingumu discusses the importance of the women’s process with other *Mamus *and makes Arhuaco authorities and political leaders aware of the role of women in the community.

Subverting static interpretations of the Law of Origin, *Mamo* Kingumwu emphasizes women’s responsibility under the *Zaku Law* (the Mother’s Law), according to which the Arhuaco woman’s body is the representation of the feminine principle of everything that exists in the universe. This imposes obligations and limitations on women, but it also leads to possibilities. In fact, constituting them as the corporeal representation of the Mother, this interpretation also turns them into the holders of a privileged political and spiritual power. Thus, the Law of Origin has become an argument that supports women’s empowerment and the revitalization of the *kunsamu*^9^, giving way to an unprecedented process of spiritual and political resistance in response to decades of violence against their traditional knowledge and practices.

Arhuaco women see this process as the possibility to guarantee their right to be recognized as political, traditional, spiritual and cultural authorities according to the Law of Origin and to participate in the direction of the CIT in issues related to health, education and human rights as well as in state scenarios such as the CEV. As expressed by one of the participants in Gunarwun:

We think that Arhuaco women have duties and rights. We have the right to be leaders, to express our opinions, to participate, to preserve our traditional and cultural knowledge and to be commissioners and *cabildas*. We want to be invited to the meetings not just to cook, but to think about how to care for the *Saku*. We have the right to be *Mamas *and to our own justice (personal communication, 2019).

### Alcira Izquierdo considers that preparing the “*ZAKU SEYNEKUN ZUN NOKWUZANAMU*

Voices of Mother Earth” report to present to the CEV provided an opportunity to cast light on Arhuaco women’s process and a means by which to demand the state to guarantee their rights:

The report is intended to ensure the protection of the rights of women who have been the victims of violence in the armed conflict and to participate in political scenarios as symbolic and spiritual representations of Mother Earth. It also highlights that we are at risk of physical and cultural extermination (personal communication, 2021).

## Methodological gaps and political tensions surrounding Arhuaco women’s participation at the CEV

Important gaps stem from the way in which Arhuaco women deal with the past and the methodological framework designed by the CEV to fulfil its task. These gaps are related to the conflicting ontologies that underlie the CEV’s knowledge-production model and Arhuaco approaches to memory and working through traumatic events. According to Mario Blaser and Marisol de la Cadena, ontological conflicts revolve around incommensurable understandings of existing entities and relationships, which communities enact through political practices and express in their collective narratives (Blaser 2013; de la Cadena 2015). Belkis Izquierdo, Arhuaco judge at the Special Jurisdiction for Peace, the judicial organ of the SIVJRNR, explains that ontological conflicts also underlie tensions between Indigenous norms and practices concerning justice, reparation and reconciliation and the dominant paradigm of transitional justice and human rights, which is embedded in anthropocentric assumptions (Izquierdo and Viaene 2018). Intra-community tensions surrounding Arhuaco women’s leadership and the CEV’s strategy to confront political issues within Indigenous communities contribute to deepening these gaps and produce further hindrances to their participation in the truth-seeking process.

### The truth is in the struggle: agency, languages and temporalities of memory

For Arhuaco women, collecting information and producing knowledge with restorative purposes, as the CEV does, requires a centring of their cosmological perspectives and organizational processes in defence of territory and Indigenous women’s rights, bringing these into dialogue with the social sciences. For the Arhuaco people remembering is no different from implementing actions to resist attempts to exterminate the Arhuaco people physically and culturally. The Arhuaco people survived the violence of the Capuchin Mission. Through weaving, speaking their mother tongue and transmitting their ancestral knowledge, women ensure the continued existence of the Arhuaco people.

This is consistent with Arhuaco epistemology, which does not distinguish the production of knowledge from its application. Moreover, the Arhuaco people hold an ethical principle of non-violence which teaches that to be able to repair and harmonize the physical and spiritual imbalances left by violent events, the violence must not be named. For them, speaking about the violence is like “sowing” words about it that will then bring more violence to the territory. During the 2019 diploma course for instance, the EIDI team observed that it was very difficult to approach the topic of sexual violence and abuse committed against girls and women at the Capuchin boarding school. Instead, the spiritual authorities spoke of the need to undertake traditional work for which Arhuaco women suggested engaging in activities related to weaving, *pagamentos* and visits to sacred sites.

According to Arhuaco participant Yesica Izquierdo, sacred sites are the places where Arhuaco women prepare to do the spiritual work assigned to them in each life cycle. For instance, they balance sexual desires by offering a *pagamento* at the Aty Nawowa Lake, located in the southeast of the Sierra Nevada. Women also use sacred materials in *pagamentos* to make spiritual offerings to correct children who do wrong and to harmonize mankind. The EIDI team used social mapping in the report to the CEV to include the damage caused by the armed conflict to sacred sites and its impact on Arhuaco women. This participatory and collaborative research methodology helps to address social phenomena through graphic representations of the territory. It is not intended to attain universal truths but to reveal each social group’s diverse experiences and interpretations of its territory and the internal and external tensions among the participant communities (Paulston 2000). The Arhuaco women agreed for us to use this methodology, as it makes it possible to honour their cosmological reference points and centre their priorities and agendas.

**Fig. 2:**
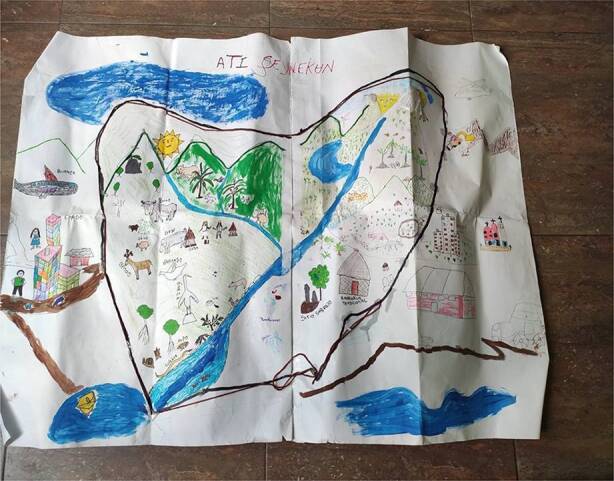
Cartographic representations of Arhuaco sacred sites. (*Intercultural School for Indigenous Diplomacy (EIDI))*

Fig. 2 shows the social map drawn up with the participants on this occasion. They divided themselves into small working groups of women of different ages and collectively created various representations of the Arhuaco territory. The participants then listed the places they consider most relevant according to their personal and collective experiences. The map shows their identification of the sacred hills, the *Kankurwas*, sacred water bodies and important community settlements. Reflecting Arhuaco women’s process and the concerns they express in agreement with Arhuaco cosmology, this exercise gave rise to stories about the cultural damage to midwifery caused by mining activities in the region during the armed conflict. Due to environmental degradation, the materials they require to practice midwifery are no longer available at the sacred sites. Women also mentioned that they are now restricted from entering some sacred sites in which they used to dance, weave and perform spiritual rituals, as these locations are now owned by non-Indigenous people. Given the traditional roles that Arhuaco women play in the community, this damage affects the entire Arhuaco population.

Arhuaco women deal with traumatic memories by means of traditional practices and forms of agency that strengthen feminine knowledge and their role in Arhuaco self-government. This reflects their commitment to the Arhuaco principle of non-violence and the unity of theory and practice in Arhuaco epistemology. As most truth commissions, the CEV, in contrast, approaches the construction of truth and memory by privileging the factual reconstruction of violent events. To clarify patterns of violence, the CEV gathers descriptions presented by people in their testimonies to the Commission and contrasts them with other sources such as official documents or academic literature. Accordingly, reports submitted to the CEV are expected to present a “description and analysis of a specific aspect of the armed conflict” (Comisión para el Esclarecimiento de la Verdad and la Convivencia y la No Repetición 2018a). During a workshop on transitional justice and historical memory, Yuirwai Tórres stated the following:

In the community, we are tired of institutions coming to ask for painful information. To construct the memory of violence committed against Arhuaco women, the *pagamentos* and the traditional work that women’s processes foster are crucial. We cannot talk about this pain in any way (personal communication, 2019).

At the second meeting with CEV lead researchers, we explained the report’s focus on Arhuaco women’s practices of cultural and epistemic resistance as potential sites for truth-finding. Besides research nuclei dealing with the actors and dynamics of the armed conflict, an independent unit at the CEV investigates coping and non-violent resistance strategies and peaceful transformations. Within the research nucleus on the causes, dynamics and impact of the armed conflict on ethnic peoples led by the EPD, another independent research axis is devoted to resistance practices. In response to our claim, one of the CEV researchers suggested that our approach could feed into the Commission’s investigation on resistance strategies by “illuminating the tensions and challenges that Arhuaco women face in mixed spaces, among other difficulties” (personal communication, February 24, 2020). A gap between Arhuaco approaches to past violence and the work of the Truth Commission is evident in the fact that even research on resistance practices highlights difficulties and victimization. The separation between the clarification of the truth about violence in the past and present agency to deal with its effects—inherent to the CEV’s epistemological framework—does not admit that it is possible to find truth and construct the memory of violent events as traditional knowledge and political processes are strengthened. As the EIDI team presented the report to the community in Umuriwa, Major Vicente, an Arhuaco traditional authority, connected this concern with the pending tasks of the implementation of the peace agreements in Indigenous territories^10^: “if they are going to keep asking us where it hurts when there is no medicine, we will not believe them” (personal communication, 2020).

According to the CEV’s Knowledge Director, the Commission’s methodology is mainly “participative” (personal communication, June 5, 2020). However, similar to previous research and truth commissions, the CEV’s research model relies on epistemological assumptions and historical perspectives characteristic of Occidental modernity that do not recognize Indigenous cosmologies and the methodologies for knowledge production these entail. As expressed by Leidy Pacheco, Indigenous women’s representative at the CEV:

In the plenary debates, ethnic commissioners find it hard to position issues related to the role of spirituality for Indigenous communities […] if it is not quantifiable, if you cannot demonstrate it, then, it is very hard to convince the plenary that it is important. Regarding sexual violence, we know that it reflects on Indigenous women’s relationship with the territory, but it is difficult to translate that into figures, as quantitative research demands. These are issues where we don’t have many resources to draw from in order to construct a pattern. Moreover, Indigenous people’s time is not linear; in our view, the time before us is the time that already passed. But they ask on what we base that, or how we can prove it. They argue that answering that it is true from the perspective of this or that particular cosmology is not enough (personal communication, March 11, 2020).

In these circumstances, we question the real scope of the CEV’s participation strategy as pertains to Indigenous women and their organizing process. We thought Indigenous women would have the opportunity to participate in the peace process, that the CEV would take their methodologies seriously and recognize Arhuaco women as epistemic subjects. But especially the National Commission of Indigenous Women saw that the Truth Commission is desperate to locate cases and construct patterns of violence. That perspective is alien to Indigenous women’s processes.

Arhuaco women also highlight that the gap between the ways the CEV and the Arhuaco people understand and deal with the past is related to the incommensurable languages in which these processes take place. So far, the activities of the Arhuaco women’s process have consisted in spiritual rituals taking place at the *Kadukwu*, where women converse with and listen to Mother Earth in the *Iku* language, without mediation of any formal systematization process (Quigua 2017). According to the CEV’s ethnic methodology, “the ethnic approach is guided by the principle of recognizing cultural diversity and its methodologies value ethnic peoples’ forms of production, narration and diffusion of knowledge and experiences through cultural and artistic expressions that display their cosmologies” (Comisión para el Esclarecimiento de la Verdad and la Convivencia y la No Repetición 2018b, p. 12). However, as expressed by a staff member of the EPD, “due to the CEV’s time and budget limitations, in many cases, it is only possible to hold the collective interview with an Indigenous community and nothing else” (personal communication, September 8, 2020). According to the EPD Director, the Covid-19 pandemic also affects the Commission’s work with ethnic peoples since most communities do not have access to Internet or electricity, and, in this new context, the Commission is no longer a priority for them (personal communication, June 16, 2020).

In 2020, the CEV worked with an Arhuaco team to document the dynamics of the armed conflict taking place in the Arhuaco territory. However, the aforementioned circumstances preclude a sustained process of interaction and trust-building with Arhuaco women in their usual environments that could help comprehend and honour the facts and interpretations they express in their political struggles and everyday traditional activities. Although the Commission values cultural expressions of ethnic peoples such as rituals and other traditional activities, the methodological approach it implements does not allow the validation of these practices as proper clarification sites. Moreover, Arhuaco women note the time lag that exists between their process and the CEV’s timeline. Initially, the CEV expected to receive all reports by the end of 2019 and must present a final report at the end of its three-year term of office in 2021. The spiritual litigation process with Mother Earth in which Arhuaco women are engaged has required almost half a decade of reflection, *pagamentos* and other traditional methodologies, and will require at least half a decade more. Only after this period will they be prepared to fully engage in self-government and participate directly in institutional contexts.

### Intra-community tensions surrounding Arhuaco women’s leadership and the CEV

**Fig. 3:**
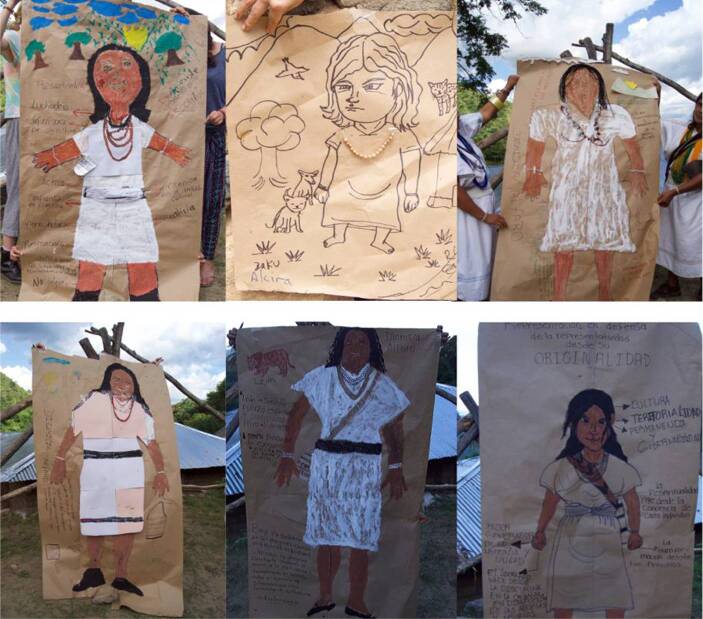
Cartographic representations of Dionisia Alfaro. (*Intercultural School for Indigenous Diplomacy (EIDI))*

In one of the diploma courses, the EIDI team and Arhuaco women participated in a body mapping activity inspired by the book *Dionisia: Autobiography of an Arhuaco Leader *(Alfaro and Jaramillo Toro 2020). The book tells the story of an Arhuaco woman who was abducted by the Capuchin Mission when she was a girl but later became an important leader and advocate of Arhuaco culture. Body mapping consists in creating life-size images of the human body through drawings, paintings and other art-based techniques (Gastaldo et al. 2012). In this case, the team used this methodology to show and tell Dionisia’s life story and for the participants to recreate Arhuaco women’s leadership in a visual and creative way. Members of the EIDI team read fragments of the book in which Dionisia talks about her childhood in the Mission and the challenges she faced as a female leader of the Arhuaco people. The participants then created body maps that represent Dionisia in her traditional dress with her weaving materials and seed necklaces. They also painted the traditional spindle that Arhuaco women use to weave and the clay pot or *poporo *that represents the Arhuaco woman. Fig. 3 presents the results of the activity.

While drawing these body-maps, the participants told stories they had heard about Dionisia and reflected on their everyday lives. Seydin Rosado commented that “the Mission prohibited the main Arhuaco rituals such as the traditional baptism, marriage and mortuary, and it disregarded the traditional knowledge system of Arhuaco families, *Mamus *and authorities. The Mission forcefully imposed the Catholic rites of first communion and marriage on the interned Arhuaco children, with the marriage rite being a condition for them to leave the Mission” (personal communication, 2019). Another participant expressed that “even though Dionisia endured the Mission’s prohibitions of weaving, speaking *Iku* and doing *pagamentos*, as well the physical punishment inflicted by the priests, she fought to preserve her culture to the end of her days” (personal communication, 2019). One of Dionisia’s sons, Cabildo governor of Businchama, told the group that, as a leader, his mother often felt forced to choose between her family and her community, which is why some members of the community discredited her leadership: “My mother was very committed, she always attended meetings and supported our processes. We grew up with our aunts and grandmothers because she was totally devoted to the struggle” (personal communication, 2019).

This activity took place at the Assembly House in Gunarwun, the community’s political and justice centre. Sowing Dionisia’s story at this site had a symbolic impact on the legitimacy of the women’s process in the Arhuaco community. The Arhuaco women’s process is supported by the spiritual authorities of the Arhuaco people. As expressed by Alcira Izquierdo: “The *Mamus *and the communities chose me to lead this process, and I work under their guidance. Our process is supported by our husbands and children” (personal communication, 2019). However, some authorities do not trust the process, as they consider it a risk for the community’s unity and autonomy as the central axis of the Arhuaco political agenda. Other members of the Arhuaco people consider that the women’s process responds to external or Western influences on Indigenous communities and argue that it is not relevant for the current situation of the Arhuaco people. As expressed by Leonor Zalabata, a member of the Arhuaco government, for instance: “From the current Board of Directors we have not seen results, nor do we fully understand what the women’s process consists of. Our tradition speaks of people and not of internal distinctions between men and women” (personal communication, May 16, 2017).

Those who disregard the women’s process are male and female leaders who usually represent the Arhuaco people in contexts of dialogue with the government and NGOs. They reserve themselves the right to participate in these scenarios and are not interested in making the Arhuaco women’s process visible. In fact, the members of the Arhuaco team in charge of interfacing with the CEV until 2020 were human rights leaders who did not recognize the women’s process. They requested the Truth Commission to prioritize the clarification of the assassination of three leaders of the Arhuaco Board of Directors in 1990 and to make this an emblematic case of violence against Indigenous people. As expressed by one of the members of the documentation team: “after several months of documentation, it is difficult for the women’s case to find a foothold in this space” (personal communication, October 10, 2020).

As an intercultural team we observe a relationship between this intra-community tension, which is also present in other Indigenous groups, and the reluctance of the CEV to coordinate with the National Commission of Indigenous Women on the clarification objectives and procedures concerning Indigenous women. At prior consultation with Indigenous peoples, the CNMI asked for woman, family and generation coordination within the EPD. In turn, the CEV hired an Indigenous women’s representative from the CNMI. However, she does not have sufficient budget or autonomy to work with Indigenous women in their territories and implement the woman, family and generation approach in the clarification activities. Since this prior consultation, the CNMI has tried unsuccessfully to participate in the CEV’s documentation and analysis procedures.

The CNMI was created as a reparation measure linked to Constitutional Court’s Act 008 of 2009, which addresses the situation of forcefully displaced Indigenous women, and is in charge of monitoring the implementation of public policy related to Indigenous women, children and older persons in Colombia. Instead of collaborating with the CNMI to guarantee Indigenous women’s participation in the truth-seeking process and therefore strengthening the CNMI as Indigenous women’s legitimate representative body, the CEV’s strategy has been to collect Indigenous women’s testimonies individually. As expressed by Lejandrina Pastor, woman, family and generation coordinator at ONIC and commissioner at CNMI, this has resulted in “the CEV possibly having made progress with Indigenous peoples, but not with Indigenous women. Indigenous women’s issues are simply obsolete in the work of the CEV. Unless women are assigned their own budget and are actively consulted, they will be left out” (personal communication, August 29, 2020).

Ignoring the CNMI weakens its autonomy and limits its reparative potential for Indigenous women. The lack of coordination between the CEV and the CNMI has precluded the possibility for Indigenous women themselves to contribute to bridging the methodological gaps the Arhuaco women perceive. Hence, although the Truth Commission has carried out some documentation processes with Indigenous organizations and has hired staff for this purpose, there is no guarantee that Indigenous women are able to participate or that the CEV implements the gender, woman, family and generation approach agreed upon in the Ethnic Chapter.

## Conclusions

The gaps between Arhuaco women’s approaches to memory and ways to deal with the past and the CEV’s methodological framework render the Arhuaco women’s political process and the truths it contains challenging. The interplay between intra-community tensions and the CEV’s strategy to confront them further contributes to relegating Arhuaco women within the Commission’s narrative and may obscure their struggles and resistant agencies behind the representation of their victimization. The CEV undoubtedly builds on important lessons learnt from the experience of previous truth commissions regarding the incorporation of differential approaches and interfacing with representatives of diverse social sectors to guarantee their participation. However, these findings suggest that, as in the case of Peru and Guatemala, additional measures are required in order to write a national narrative that is “more inclusive of groups that are historically marginalized within the nation-state” (Theidon 2006, p. 457), such as Arhuaco women in Colombia.

Methodological gaps such as the ones we analyze in this article show that incorporating an intersectional perspective in truth-seeking processes, and therefore connecting factors such as language, ethnicity and culture to the analysis of gender, which the PTRC failed to do, requires the development of methodological approaches embedded in sustained processes of trust-building and knowledge exchange that centre Indigenous women’s cosmologies and practices. The case of Arhuaco women shows that such approaches may involve finding alternatives to interview questionnaires that help to produce linear accounts of past victimization and allowing more extended and flexible research time frames. This would allow truth commissions to recognize them as epistemic subjects and to construct knowledge collectively with them.

The experience of Arhuaco women before the CEV also highlights that another obstacle for Indigenous women’s full participation in truth-seeking processes are Indigenous leaders who, according to “orthodox” interpretations of traditional laws, continue to claim that Indigenous women may not interfere with state institutions, as well as truth commissions that do not recognize the autonomy of Indigenous women’s representative bodies. These political tensions may end up marginalizing Indigenous women’s priorities for clarification and undermining the possibility to foster exemplary social rejection of harms committed against them, as happened with the CEH. An intersectional framework would therefore be useful in guaranteeing the participation of Indigenous women in truth-seeking processes if it entails a strengthening of the processes they lead to defend their individual and collective rights and preserve their traditional knowledge and cultural practices.

Bridging methodological gaps and solving political tensions surrounding Indigenous women’s participation in truth-seeking processes are crucial steps towards fulfilling the restorative purpose of truth commissions and to respond to Indigenous women’s need for recognition, rehabilitation and reparation. Arhuaco women demand that the CEV recommendations to the Colombian government in the final report include the revitalization of their traditional knowledge through the construction of ceremonial houses, schools for spiritual formation and productive projects for economic relief, as well as public apologies by the Catholic Church for the damage caused by the Capuchin mission to the traditional knowledge and culture of Arhuaco people, and especially women. Very often a deeply transformative vision of transitional justice underlies Indigenous women’s requests for non-repetition. However, as in the case of the TRC, they are often not followed through. Honouring them would truly contribute to breaking the continuum of colonial and armed conflict violence in their territories and prevent further violence and human rights abuses against them.

## Abbreviations

AUC: United Self-Defence Forces of Colombia
CEH: Guatemalan Historical Clarification Commission
CEV: Truth, Coexistence, and Non-Repetition Commission
CIT: Tayrona Indigenous Confederation
CNMI: National Commission of Indigenous Women
CONADEP: National Commission on the Disappearance of Persons
CONPA: National Afro-Colombian Peace Council
EIDI: Intercultural School for Indigenous Diplomacy
ELN: National Liberation Army
EPD: Ethnic Peoples Directorate
FARC: Revolutionary Armed Forces of Colombia
GWG: Gender Working Group
ICTY: International Criminal Tribunal for the Former Yugoslavia
MPC: Permanent Roundtable with Indigenous Peoples and Organizations
ONIC: National Indigenous Organization of Colombia
PTRC: Peruvian Truth and Reconciliation Commission
SIVJRNR: Comprehensive System of Truth, Justice, Reparation, and Non-Repetition
TRC: Canadian Truth and Reconciliation Commission

## Footnotes

1 The transmission is available here: https://fb.watch/4awtc6aQiq/.

2 According to the national census of former FARC combatants, 20 individuals in the Cesar and Guajira departments recognized themselves as Arhuaco people forcefully recruited in their youth (eight women and 12 men) (Universidad Nacional de Colombia 2017).

3 *Iku *is the Arhuaco word for “people”.

4 *Mamos *are Arhuaco political and spiritual leaders.

5 *Pagamentos *are massive rituals and traditional work directed by the *Mamos*.

6 *Marneykuan* is physical work performed to honour spiritual mothers and fathers.

7 *Kankurwa *is an Arhuaco sacred house of knowledge and life.

8 *Kadukwu* is an Arhuaco sacred site in which to communicate with nature.

9 *Kunsamu are *the principles of Arhuaco life.

10 According to the Colombia Barometer Initiative led by the Kroc Institute for International Peace Studies, up to May 2020, only 10% of the provisions of the Ethnic Chapter of the peace agreements have been implemented and 9% have achieved an intermediate level of progress. The latter include provisions related to the effective participation of ethnic communities in the institutions of the SIVJRNR. In all, 49% of the Ethnic Chapter’s provisions have achieved minimal progress and the implementation of 32% of the provisions have not begun (2021).
